# Identification and characterization of multipotential stem cells in immortalized normal ovarian surface epithelial cells: The pluripotency of immortalized normal ovarian surface epithelial cells

**DOI:** 10.3724/abbs.2023253

**Published:** 2024-01-19

**Authors:** Lin Hou, Hanqing Hong, Wenjiao Cao, Liutong Wei, Lichun Weng, Shuang Yuan, Chengqi Xiao, Qiuwan Zhang, Qian Wang, Dongmei Lai

**Affiliations:** 1 The International Peace Maternity and Child Health Hospital School of Medicine Shanghai Jiao Tong University Shanghai 200030 China; 2 Shanghai Key Laboratory of Embryo Original Diseases Shanghai 200030 China

**Keywords:** ovarian surface epithelial cell, transcriptome, multipotential stem cell, oocyte, repair function

## Abstract

The ovarian surface epithelium (OSE) is a single layer of squamous-to-cuboidal epithelial cells that experience repetitive ovulatory rupture and subsequent repair. However, the characteristics of human immortalized ovarian surface epithelial cells (IOSE80) remain elusive. This study aims to determine whether IOSE80 cells have the characteristics of stem cell proliferation and multilineage differentiation and their application in regenerative medicine. IOSE80 cells are sequenced by high-throughput transcriptome analysis, and 5 sets of public data are used to compare the differences between IOSE80 cells and bone marrow mesenchymal stem cells, pluripotent stem cells, and oocytes in transcriptome profiling. The IOSE80 cells present a cobblestone-like monolayer and express the epithelial cell marker KRT18; the stem cell markers IFITM3, ALDH1A1, and VIM; lowly express stem cell marker LGR5 and germ cell markers DDX4 and DAZL. In addition, the GO terms “regulation of stem cell proliferation”, “epithelial cell proliferation”, *etc*., are significantly enriched ( *P*<0.05). IOSE80 cells have the potential to act as mesenchymal stem cells to differentiate into adipocytes with lipid droplets, osteoblasts, and chondroblasts *in vitro*. IOSE80 cells express pluripotent stem cell markers, including OCT4, SSEA4, TRA-1-60, and TRA-1-81, and they can be induced into three germ layers *in vitro*. IOSE80 cells also form oocyte-like cells *in vitro* and *in vivo*. In addition, IOSE80 cells exhibit robust proliferation, migration, and ovarian repair functions after *in vivo* transplantation. This study demonstrates that IOSE80 cells have the characteristics of pluripotent/multipotent stem cells, indicating their important role in tissue engineering and regenerative medicine.

## Introduction

The ovary is covered by a single layer of squamous, cuboidal, or columnar ovarian surface epithelium (OSE), and it plays an important role in normal ovarian physiology [ 1‒ 4]. During the lifespan, these epithelial cells can encounter a series of cell cycle events, including quiescence, mitosis, epithelial rupture, repair, apoptosis, and degeneration [ 5, 6]. As follicles are selected to develop during the follicular phase of the cycle, they are juxtaposed with the ovarian surface, and OSE cells in this region undergo mitosis to accommodate the rapid increase in follicle size. A key mechanism involved in wound repair is epithelial-mesenchymal transition (EMT) [ 7, 8]. EMT is necessary for wound repair, enabling cells to migrate and secrete ECM proteins into the injured area [9]. The OSE for morphological EMT during wound repair in rhesus monkeys and rats has been studied, in which the OSE layer is flat on the developing corpus luteum [ 10, 11]. After ovulation, cells left in the postovulatory follicle and epithelial cells undergo luteinization and form the corpus luteum (CL), a transient endocrine structure that produces progesterone or degenerates without pregnancy [12]. The recovery of OSE is very fast and effective, and the wound is completely closed within 12 h to 3 days after rupture in mice [13]. The remarkable circulation and regeneration abilities of OSE indicate the existence of resident stem cells.

In recent years, the stemness characteristics of ovarian epithelial cells have been reported [ 14, 15]. The gene expression profile supports the hypothesis that human ovarian epithelial cells are pluripotent [16]. Bowen *et al*. [16] conducted comparative gene expression profiling analysis on normal human ovarian surface and ovarian cancer epithelial cells isolated from human serous papillary ovarian adenocarcinoma. The genes related to the maintenance and pluripotency of adult stem cells are highly expressed in healthy ovarian surface epithelial cells, while they are not expressed or are expressed at low levels in serous papillary ovarian adenocarcinoma [16]. OSE was found to express several well-established markers of stem cells, including NANOG, CD44, ALDH1A1, ALDH1A2, SFRP1, and LIM homeobox proteins LHX2 and LHX9 [ 16‒ 18]. In addition, they express stem cell markers, such as LY6A (also known as SCA1), Lgr5, Procr (protein C receptor), and c-KIT (KIT ligand) [ 19‒ 22].

Moreover, OSE has the potential to differentiate into multiple cell lineages. Many previous studies demonstrated that small round-shaped cells in the upper cortex of the ovarian surface derived from women are positive for markers of pluripotent embryonic stem cells, and they could differentiate into oocyte-like cells or parthenogenetic blastocyst-like structures *in vitro* [ 23‒ 28]. In addition, our research team reported for the first time that ovarian epithelial cells could be obtained from human follicular fluid. In the *in vitro* culture system, these epithelial cells spontaneously form oocyte-like cells and express specific germ cell markers (DAZL, STELLA, *etc*.). Furthermore, these ovarian epithelial cells exhibit clonal growth, express markers of early embryonic stem cells, and could differentiate into all three germ layers [29]. Recently, Wu *et al*. [30] indicated that there are six different cell types in preovulatory follicular fluid, including epithelial cell subpopulations expressing CDH1, KRT18, and EPCAM. Although the presence of epithelial cells in follicular fluid has been demonstrated, the number of epithelial cells is very limited.

The IOSE80 cell line represents immortalized cells of the ovarian epithelium. These cells were obtained from normal human ovarian epithelial cells that had been transfected with SV-40T [31]. It not only maintains some morphological characteristics and molecular markers of normal ovarian epithelium but also has strong proliferation ability *in vitro*. It is commonly used to study the origin of epithelial ovarian cancer or as a control for epithelial ovarian cancer cell research. However, its characteristics have not yet been fully identified.

In the present study, we aimed to investigate the potential characteristics of pluripotent/multipotent stem cells in IOSE80 cells. A systematic comparison of the transcriptome profiling of IOSE80 cells, stem cells, and oocytes was conducted to identify similarities and differences. Next, experiments were performed to examine whether IOSE80 cells possess characteristics of pluripotent stem cells (PSCs) and their capability to form three germ layers. Additionally, we attempted to investigate whether IOSE80 cells could induce oocytes both *in vitro* and *in vivo*, as well as their repair function.

## Materials and Methods

### Sample collection and cell culture

Human follicular fluid was collected and cultured as described previously [29]. For epithelial cells derived from human follicular fluid (hFF-ECs), the cells were grown and cultured to 80% confluence in Dulbecco’s modified Eagle’s medium/F12 medium (Gibco, Grand Island, USA). This research was approved by the Institutional Ethics Committee of the International Peace Maternity and Child Health Hospital, and written informed consent was obtained from each participant.

### IOSE80 cell culture

OSE80 cells were then provided by Shanghai Hongshun Biotechnology Co., Ltd. (Shanghai, China). IOSE80 cells were cultured in RPMI-1640 medium (Gibco) supplemented with 10% fetal bovine serum (FBS) and 1% penicillin/streptomycin (HyClone, Logan, USA). Subculturing was performed at a 1:3 ratio every 3 days.

### Trilineage differentiation and assay

IOSE80 cells were cultured in TeSR ^TM^ maintenance media for 4 days. Subsequently, the STEMdiff ^TM^ kit (STEMCELL Technologies, Shanghai, China) was employed for one week, utilizing the Ectodermal Lineages, Mesodermal Lineages, and Endodermal Lineages kits sequentially. Afterwards, the differentiated Tri-lineage layer was selected and cultured in a dish with human amniotic epithelial cells (hAECs) as a feeder layer. The lineage layer was further cultured for 2 days to perform an immunofluorescence assay.

The lineage layer was fixed with 4% paraformaldehyde for 30 min and permeabilized for an additional 10 min with 0.1% Triton X-100 (Sigma-Aldrich, St Louis, USA). The blocking step was performed for 30 min using 5% FBS in PBS. The lineage layer was incubated with antibodies against Nestin (mouse anti-human; 1:200; Santa Cruz Biotechnology, Santa Cruz, USA), anti-Sox-17 (rabbit anti-human; 1:200; Santa Cruz Biotechnology), and brachyury (mouse anti-human; 1:200; Santa Cruz Biotechnology) for 1.5 h at 37°C. Each antibody was detected using corresponding secondary antibodies conjugated to fluorescein isothiocyanate.

### *In vitro* differentiation of IOSE80 cells into osteogenic, chondrogenic, and adipogenic lineages

For osteogenic differentiation, IOSE80 cells were seeded into 0.1% gelatin-treated 6-well plates at a density of 2×10 ^4^ cells/well. When the cells reached a confluence of 70%, the medium was replaced by osteogenic differentiation medium (OriCell®, Guangzhou, China; HUXXC-90021, 10% FBS, dexamethasone, sodium β-glycero phosphate, and ascorbic acid) and kept for 4 weeks. To assess osteogenic differentiation, Alizarin Red S staining was performed for the calcium-rich extracellular matrix.

For adipogenic differentiation, 1×10 ^4^ cells were seeded per well. The cells at high confluence were then treated with adipogenic differentiation medium (OriCell®, that is, 10% FBS, IBMX, dexamethasone, insulin, and indomethacin) for 4 weeks. The cells were fixed with 4% formaldehyde solution, and lipid droplets of the resultant differentiated cells were detected by Oil Red O staining.

For chondrogenic differentiation, a Chondrogenesis Differentiation kit (OriCell®) was used according to the manufacturer’s protocol. Briefly, 1.6×10 ^7^ cells were resuspended in MSC medium (mainly TGF-β3, sodium pyruvate, ascorbate, proline, dexamethasone, ITS, *etc*.). Micromass cultures were generated by seeding 200 μL cell suspension into a 15 mL centrifuge tube with the cap released. After 2 h of micromass culture, warm chondrogenesis media were added and incubated at 37°C with 5% CO _2_. After induction of differentiation for 4 weeks, the cells were fixed with 4% formaldehyde solution and then stained with Alcian blue.

### Isolation and culture of murine granulosa cells

Ovaries from 21-day-old ICR female mice were dissected free of fat, bursa and oviduct. After the pooled ovaries were washed with F12 (DMEM/F12), the granulosa cells were released by puncturing the ovaries manually with 25-gauge needles. Cell suspensions were then passed through a 40-μm nylon cell strainer (Corning Falcon, New York, USA) and centrifuged at 200 *g* for 5 min. Pelleted cells were reconstituted in DMEM/F12 (DMEM; Life Technologies, Carlsbad, USA) supplemented with 10% FBS, 1 M NEAA, 1% penicillin/streptomycin (HyClone) and 1% ITS (100×). For *in vitro* differentiation of IOSE80 cells, GCs were treated with mitomycin C (10 mg/mL; Sigma-Aldrich) at the second passage for 2‒3 h, washed with PBS, and then plated in a culture dish precoated with 0.1% (w/v) gelatin.

### *In vitro* induction of oocyte-like cells

First, IOSE80 cells were cultured in medium without growth factors with hanging drops at 30 μL per drop for 2 days and placed under the lid of a 10-mm petri dish containing 15 mL of PBS. After 2 days, cells were collected and resuspended in medium containing 10 ng/mL bFGF (PeproTech, Cranbury, USA), 10 ng/mL bone morphogenic protein-4 (PeproTech), and 0.1 μmol L-1 RA (Sigma-Aldrich), and they were then cultured in a mitomycin C-treated granulosa cell monolayer for 4‒5 days. The medium was changed every 2 days. Finally, the cells were cultured in differentiation medium containing BMP-4 (10 ng/mL; PeproTech), EGF (10 ng/mL), bFGF (10 ng/mL), ITS (100×), pregnant mare serum gonadotrophin (PMSG; 1 IU/mL), human chorionic gonadotropin (hCG; 1 IU/mL), estrogen (1 ng/mL), progesterone (1 ng/mL), and human follicular fluid. The medium was changed every 2 days. Every 5‒7 days, cells were transferred onto a freshly treated granulosa cell monolayer. This process was performed for 25‒30 days.

### Immunofluorescence staining

IOSE80 cells, oocyte-like cells, and colonies maintained on hAECs were fixed with 4% paraformaldehyde for 15‒20 min at room temperature and permeabilized with 0.1% Triton X-100 for 10 min at room temperature. Cells were then blocked with 5% FBS in PBS for 30 min and incubated with primary antibodies, including anti-NANOG (rabbit anti-human; 1:200; Abcam, Cambridge, UK), anti-EpCAM (goat anti-human; 1:500; Santa Cruz Biotechnology), anti-IFITM3 (rabbit anti-human; 1:200; Novus Biologicals, Littleton, USA), anti-ZPC (rabbit anti-human; 1:200; Santa Cruz Biotechnology), anti-SCP3 (rabbit anti-human; 1:800; Abcam), anti-GDF9 (rabbit anti-human; 1:200; Millipore, Billerica, USA), anti-SSEA4 (mouse anti-human; 1:100; Millipore), anti-Tra-160 (mouse anti-human; 1:100; Millipore), and anti-Tra-1-81 (mouse anti-human; 1:100; Millipore) antibodies for 1 h at room temperature. Cells were then probed with fluorescein isothiocyanate-labelled IgG (1:200; Santa Cruz Biotechnology) or rhodamine (TRITC)-labelled IgG (1:100; Invitrogen, Carlsbad, USA) antibodies and incubated at room temperature for an additional 30 min. Each antibody was detected using corresponding secondary antibodies conjugated to fluorescein isothiocyanate. Fluorescence images were obtained using a Leica DMI3000 microscope or an SP8 confocal microscope (Leica Microsystems, Wetzlar, Germany).

### Western blot analysis

Proteins were extracted from IOSE80 cells using RIPA lysis buffer (Yeasen, Shanghai, China) supplemented with protease inhibitor cocktail (Yeasen) on ice. Cell lysates were cleared via centrifugation at 20,913 *g* for 15 min. The protein concentration was measured using the BCA kit (Thermo Fisher Scientific, Waltham, USA). Proteins were separated by 10% or 15% sodium dodecyl sulfate-polyacrylamide gel electrophoresis (SDS-PAGE) and transferred onto polyvinylidene difluoride (PVDF) membranes (Millipore), and the membranes were then blocked with blocking solution at room temperature for 1 h, and then incubated with rabbit antibodies against vimentin (1:1000; CST, Beverly, USA) and tubulin (1:1000; CST) overnight at 4°C. The membranes were incubated with horseradish peroxidase-conjugated secondary antibodies (1:2500; CST) for 1 h at room temperature, and the bands were detected using an enhanced chemiluminescence system (ECL; Millipore).

### Flow cytometric analysis

The manufacturer’s instructions (MULTI SCIENCES, Hangzhou, China) were followed. A cell suspension was collected, and the number of cells ranged from 2×10 ^5^ to 1×10 ^6^ cells/mL. The cells were then centrifuged, and the supernatant was removed. PBS was used to wash the cells once, and the supernatant was discarded. Then, 1 mL of reagent A and 10 μL of reagent B were added to the cells, which were vortexed and mixed for 5‒10 s. The mixture was incubated at room temperature for 30 min. Finally, the percentages of cell cycle distribution were evaluated through PI staining, and all samples were analyzed using FACSCalibur (BD, Franklin Lakes, USA) with appropriate software (ModFit LT; BD).

### Wound healing assay

IOSE80 cells were seeded into 6-well plates, and a 200-μL pipette tip was used to create scratch wounds when the cells reached high confluence. After wash with PBS, the cell culture medium was replaced by 10 μg/mL mitomycin C in FBS-free RPMI-1640 medium to weaken the interference effects on cell proliferation. The healed wounds were photographed at 0, 12, and 24 h after scratching.

### EGFP-containing lentiviral infection

After IOSE80 cells were cultured for 2 days, lentiviral infection was performed using eGFP lentivirus (GM100101-1; Genomeditech, Shanghai, China) according to the manufacturer’s instructions.

### Immunomagnetic sorting

The Eppendorf tube containing IOSE80 cells and magnetic beads was placed on the magnetic separation rack and mixed for 2‒3 min. Then PBS was removed, and the Eppendorf tube was removed from the rack. Next, PBS was added to slowly resuspend the cells, followed by another round of magnetic separation. Finally, IFITM3-positive cells were used for culture or cell transplantation.

### Xenotransplantation of IOSE80 cells

IOSE80 cells were harvested at 48 h after lentiviral transfection. Next, the cells were washed thrice with PBS, collected, and resuspended in PBS. For transplantation I, IOSE80 cells carrying GFP (50 μL, containing 1.3×10 ^5^ cells) were transplanted into murine ovaries *in vitro* ( *n*=10, ICR, 6 weeks old; Shanghai SLAC Laboratory Animal Co. Ltd, Shanghai, China), and the ovaries were then transplanted subcutaneously into NOD-SCID mice ( *n*=10, 8 weeks old, female; Shanghai SLAC Laboratory Animal Co. Ltd ). For transplantation II, IOSE80 cells transfected with GFP were transplanted. The cells were sorted by using IFITM3 antibody, and the IFITM3-positive cells (50 μL, containing 1.3×10 ^5^ cells) were then transplanted into the murine ovaries *in vitro* ( *n*=10, ICR, 6 weeks old). Thereafter, the ovaries were transplanted into the subcutaneous tissue of NOD-SCID mice ( *n*=10, 8 weeks old, female). After 14 days, xenotransplantation ovaries were taken from nude mice subcutaneously for immunofluorescence evaluation of the differentiation of IOSE80 cells *in vivo*.

### Assessment of ovarian repair function

A group of twenty 8-week-old ICR mice were administered with 5 IU pregnant mare serum gonadotropin (PMSG) via intraperitoneal (i.p.) injection, followed by injection with 5 IU human chorionic gonadotropin (hCG; SANSHENG Biological Technology, Ningbo, China). The hCG injection was given at 44‒46 h after the PMSG injection. IOSE80 cells carrying GFP were collected and transplanted into the ovaries of mice (50 μL containing 5×10 ^5^ cells) at 16 h after the injection of hCG. The murine ovaries were collected on days 3 and 7 after cell transplantation. Immunofluorescence technique was employed to investigate the tissue compatibility of IOSE80 cells expressing GFP on day 3 and 7 post-transplantation.

### Transcriptome analysis

Total RNA was extracted using Trizol magnetic bead method(Majorbio, Shanghai, China) , the concentration and purity of the extracted RNA were detected with NanoDrop 2000, the integrity of RNA was detected by agarose gel electrophoresis, and the RIN value was determined with Agilent 2100 (Agilent Inc., Santa Clara, USA).

To establish a single database, a total RNA amount of ≥1 μg and a concentration of ≥35 ng were needed, with an OD260/280 ≥1.8 and an OD260/230 ≥1.0. The eukaryotic mRNA 3′ end contains a polyA tail structure that can be isolated for transcriptome analysis using magnetic beads with oligo (dT) pairing with polyA for the A-T base. The Illumina NovaSeq 6000 platform was used for the sequencing of short fragments. To randomly interrupt the enriched mRNA, fragmentation buffer was added to break it into small fragments of approximately 300 bp that could be isolated through magnetic bead screening. The fragmented mRNA consisted of a comprehensive RNA sequence, with an average length of several kilobases.

### Data processing and statistical analysis

The transcript data of oocytes and stem cells in this study were downloaded from the Gene Expression Omnibus (GEO) repository. In brief, the GV and MII data were obtained from the young group dataset (GSE95477). The bone marrow mesenchymal stem cell data were derived from the BM-MSC control group dataset (GSE105145). PSC data, comprising embryonic stem cells and induced PSCs, utilized H1-hESCs (GSM3400598), H9-hESCs (GSM3400599), and iPSCs (GSM3400600). The Majorbio Cloud platform was utilized for data analysis, and the software and database used are listed in Supplementary Table S1.

Data are presented as the mean±standard deviation (SD). Statistical analysis was conducted using SPSS 22.0 software (IBM, Armonk, USA), employing one-way analysis of variance (ANOVA), followed by Tukey’s honestly significant difference (HSD) post hoc test. *P*<0.05 was considered statistically significant.

## Results

### The IOSE80 cell line has the characteristics of ovarian epithelial cells

**Figure 1:**
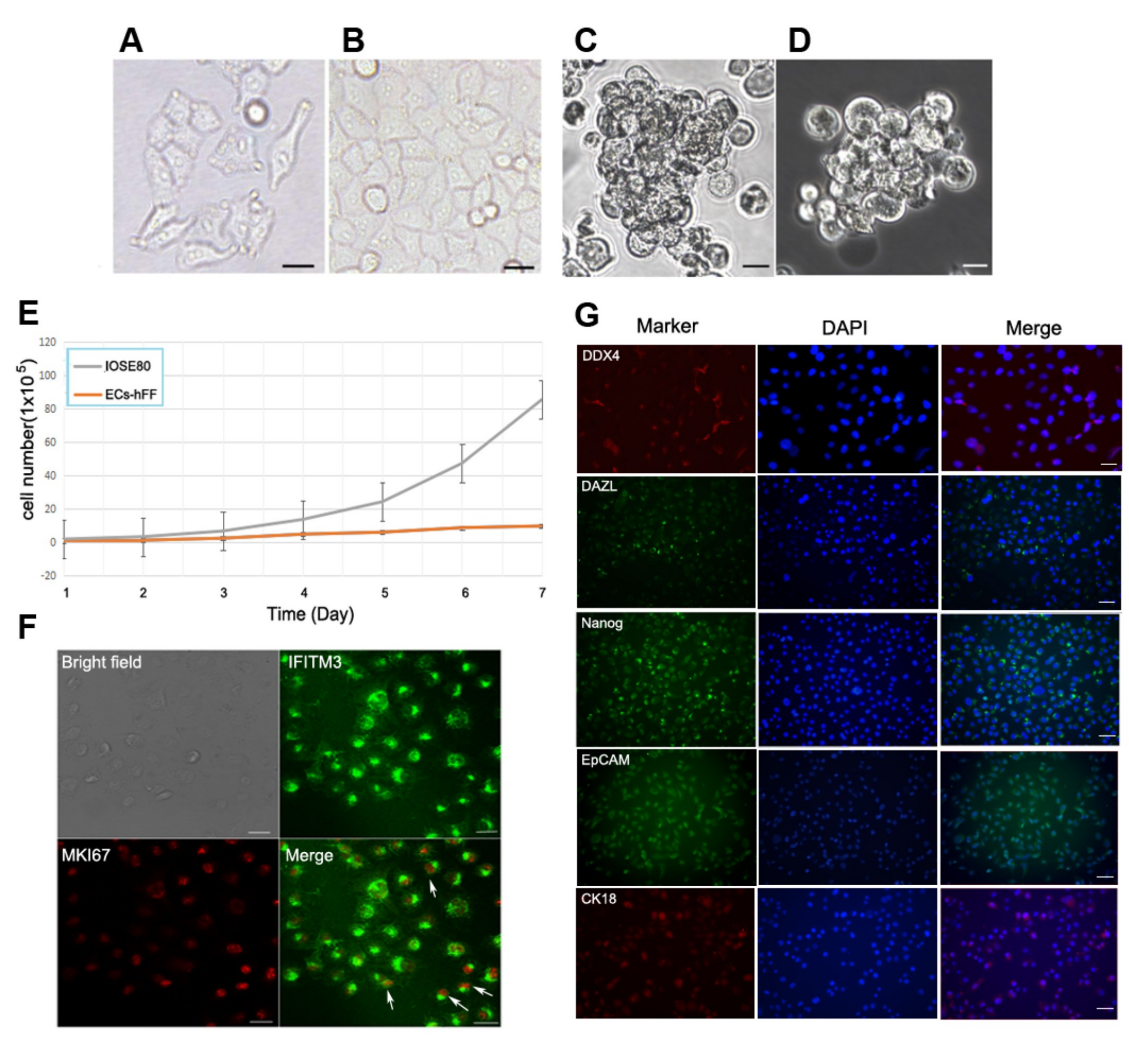
Figure 1 Characteristics of the IOSE80 cell line Cell morphology of IOSE80 cells under nonconfluent (A) and confluent (B) conditions. (C) Under low-adhesion culture conditions, IOSE80 cells become spheroids. (D) In the stem cell culture system, IOSE80 cells become spherical under a phase-contrast microscope. (E) The cell growth curve of IOSE80 cells and EC-hFF. (F) Immunostaining of IFITM3 and MKI67 in IOSE80 cells. IFITM3 (known as fragilis) shows membrane and cytoplasm staining, and MKI67 shows nuclear staining. Arrows show double-labelled cells after merging. (G) Markers of germ cells (DDX4, DAZL), stem cells (Nanog), and epithelial cells (EpCAM, CK18) are expressed in IOSE80 cells. Scale bar: 20 μm. DAPI, 4′,6-diamidino-2-phenylindole. EC-hFF, epithelial cells derived from human follicular fluid.

The morphological characteristics of immortalized ovarian surface epithelial cells (IOSE80 cells) were found to vary with changes in cell density and culture conditions during *in vitro* experiments. Initially, IOSE80 cells were cultured in RPMI-1640 medium using an adherent method. During the nonconfluent stage, typical epithelial cell-like features, such as squamous, cuboidal or columnar shapes, were observed in most IOSE80 cells. However, some cells exhibited a fibroblast phenotype, in addition to pseudopods and round-shaped cells of varying sizes ( Figure 1A). However, when the cells were confluent, they presented a cobblestone arrangement of cuboidal epithelial-like monolayers ( Figure 1B). In nonadhesive culture or stem cell culture systems, IOSE80 cells formed spheroids ( Figure 1C,D). It was previously reported that ovarian epithelial cells could be derived from human follicular fluid [29]. Compared with the primary ovarian epithelial cells derived from human follicular fluid (EC-hFF), the number of IOSE80 cells increased more rapidly with an estimated doubling time of 18-20 h, whereas EC-hFF required passage at a confluence of every 5-6 days ( Figure 1E).

The characteristics of germ cells, stem cells, and proliferation of IOSE80 cells were identified, and immunofluorescence staining showed that IFITM3 (Fragilis) was distributed in the cell membrane and cytoplasm, while Ki-67 was located in the cell nucleus and the double-stained cells represented the characteristics of proliferative germ cells ( Figure 1F). Similarly, immunofluorescence staining indicated that IFITM3 was distributed in the cell membrane and cytoplasm, while Oct4 was located in the cell nucleus and the double-stained cells represented the characteristics of stem cells ( Supplementary Figure S1A‒C). Immunofluorescence staining revealed that ALDH1A1 was distributed in the cytoplasm, while PCNA was located in the cell nucleus and the double-stained cells represented the characteristics of proliferative stem cells ( Supplementary Figure S1D‒F). In addition, IOSE80 cells expressed epithelial marker (EpCAM) and cytokeratin 18 (CK18/KRT18), germ cell marker (DDX4, DAZL), and stem cell marker (Nanog), suggesting that the IOSE80 cell line had the characteristics of ovarian epithelial cells ( Figure 1G).

### Comparative transcriptome profiling analysis of IOSE80 cells, stem cells, and oocytes

To comprehensively analyze the molecular characteristics of IOSE80 cells, RNA-Seq of IOSE80 cells was performed, and a total of 24.63 Gb clean data were obtained. The clean data of each sample were above 6.0 Gb, and the Q30 base percentage was above 94.68%. A total of 26,140 genes were detected, including 25,961 known genes and 179 new genes. In addition, 111,458 transcripts were expressed, encompassing 103,637 known transcripts and 7821 new transcripts.

High-throughput sequencing results showed that the expression level of MT-ATP8 was the highest in IOSE80 cells. Other highly expressed genes included the ovarian epithelial stem cell gene ( *ALDH1A1*), germ cell express genes ( *IFITM3)*, cytoskeleton ( *ACTB* and *VIM*), and metabolism-related enzymes ( *GAPDH* and *ALDOA*) ( Supplementary Figure S2A). Meanwhile, the transcriptome results revealed that Yamanaka reprogramming factor genes ( *Oct-3*/ *4*, *Sox2*, *Klf4*, and *c-Myc*), *NANOG*, *LIN28A*, other stem cell-related genes, and germ cell-related genes ( *DDX4*, *DAZL*, *FIGLA*), as well as hormone receptor genes ( *FSHR*, *ESR1* and *LHCGR*), were expressed at low levels in IOSE80 cells. In contrast, epithelial cell-related genes ( *EMP1* and *KRT18*) were highly expressed in IOSE80 cells ( Supplementary Figure S2B).

**Figure 2:**
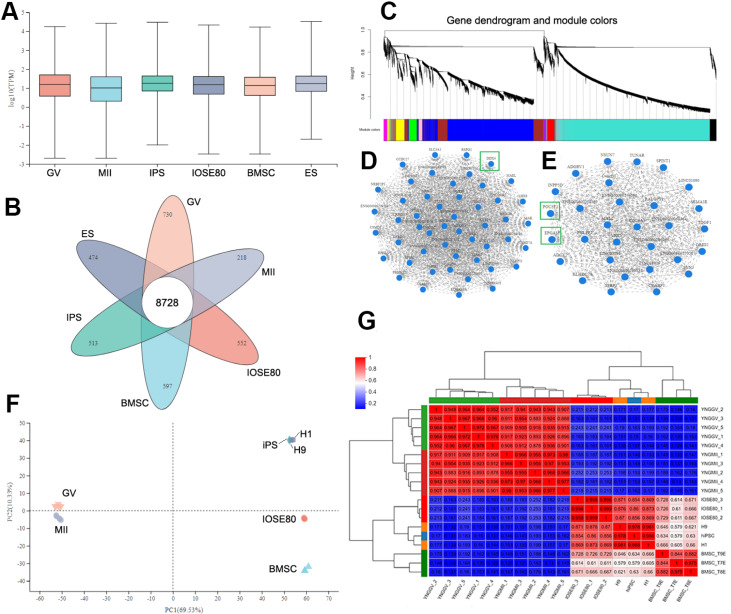
Figure 2 Comparative transcriptome profiling analysis of IOSE80 cells, stem cells and oocytes (A) Expression distribution. The abscissa is the name of the sample group, and the ordinate is the log10 value at the gene/transcript expression level. Each color box in the figure represents a group, and the horizontal line in the figure represents the median of gene/transcript expression in each group. BMSC, bone marrow mesenchymal stem cells; IPS, induced pluripotent stem cells; ES, embryonic stem cells; GV, fully grown germinal vesicle oocytes; MII, metaphase of second meiosis oocytes. (B) Venn diagram analysis shows that 6 types of cells share 8728 gene expression. The petal region data represent the unique gene expression of each type of cell. For example, IOSE80 cells have 552 genes that are not expressed in other cells. (C) WGCNA. According to the gene expression trend, genes are divided into modules, in which a branch represents a gene and a color represents a module. (D) Subnetwork of germline cell characteristics. The green box indicates the germ cell marker DDX4. (E) Subnetwork of stem cell and epithelial cell characteristics. Green boxes indicate the stem cell marker POU5F1 and the epithelial cell marker EPCAM. (F) PCA. The distance between each sample point represents the distance between samples. The distance between each sample point represents the distance between samples. The closer the distance is, the higher the similarity between samples, and the better the biological duplication between samples. The abscissa represents the contribution of principal component 1 (PC1) in the two-dimensional graph to the distinguishing samples, and the ordinate represents the contribution of principal component 2 (PC2) in the two-dimensional graph to the distinguishing samples. (G) Correlation between samples. The right and lower sides in the figure are sample names. The left and upper sides show the clustering of each sample. As shown in the color bar, the darker the red color is, the higher the correlation between the two samples, and the R2 value is shown in the figure. The P values between all pairwise samples are less than 0.001 (P<0.001).

To further determine the characteristics of IOSE80 cells, we attempted to compare the transcriptome of IOSE80 cells with bone marrow mesenchymal stem cells (BMSCs), PSCs (induced PSCs, IPS, H1 and H9), and oocytes (GV oocytes and MII oocytes). Data on the transcriptomes of other types of cells compared with IOSE80 cells were downloaded from the GEO repository. The expression distribution showed that the median gene expression of IOSE80 cells was similar to that of other types of cells, suggesting that the gene expression levels of these different types of cells were similar and appropriate for comparative analysis ( Figure 2A). Venn diagram analysis revealed that 6 types of cells shared 8728 genes, and IOSE80 cells had 13,875 genes (TPM>1) ( Figure 2B).

Weighted gene coexpression network analysis (WGCNA) was carried out on IOSE80 cells, along with BMSCs, embryonic stem cells, induced PSCs, GV-stage oocytes, and MII-stage oocytes, to investigate the similarity of the expression patterns of IOSE80 cells with those of stem cells and germ cells ( Figure 2C). Each module was analyzed, and a subnetwork composed of germline-related genes, such as *DDX4*, *MAEL*, *LHX8*, and *MA*X, was found in the turquoise module, suggesting that this network is related to germ cell characteristics ( Figure 2D). In the black module, a subnetwork composed of marker genes of stem cells and epithelial cells, such as *POU5F1* and *EPCAM*, could be identified, suggesting that this subnetwork is related to the characteristics of epithelial cells and stem cells ( Figure 2E). Coexpression analysis suggested that IOSE80 cells possess stem and germ cell marker genes, endowing them with identity characteristics similar to those of stem and germline cells. The interaction between these marker genes and other genes in the module plays an important role in the characteristics of IOSE80 and provides a molecular basis for the differentiation of IOSE80 cells into stem and germline cells. PCA showed that IOSE80 cells, BMSCs, PSCs, GV oocytes, and MII oocytes were distributed at different locations, reflecting that they represent different cell types ( Figure 2F). IPS, H1, and H9 cells in PSCs were clustered together, demonstrating that these three types of cell transcriptomes are similar and may be classified into one group; similarly, the three samples of IOSE80 cells were the closest to each other after dimensionality reduction analysis, indicating that the three samples of IOSE80 cells have the highest similarity and reproducibility. Notably, gene expression correlation analysis revealed that the expression cluster of IOSE80 cells was closer to PSCs than to BMSCs ( Figure 2G).

### The IOSE80 cell line has the characteristics of BMSCs

**Figure 3:**
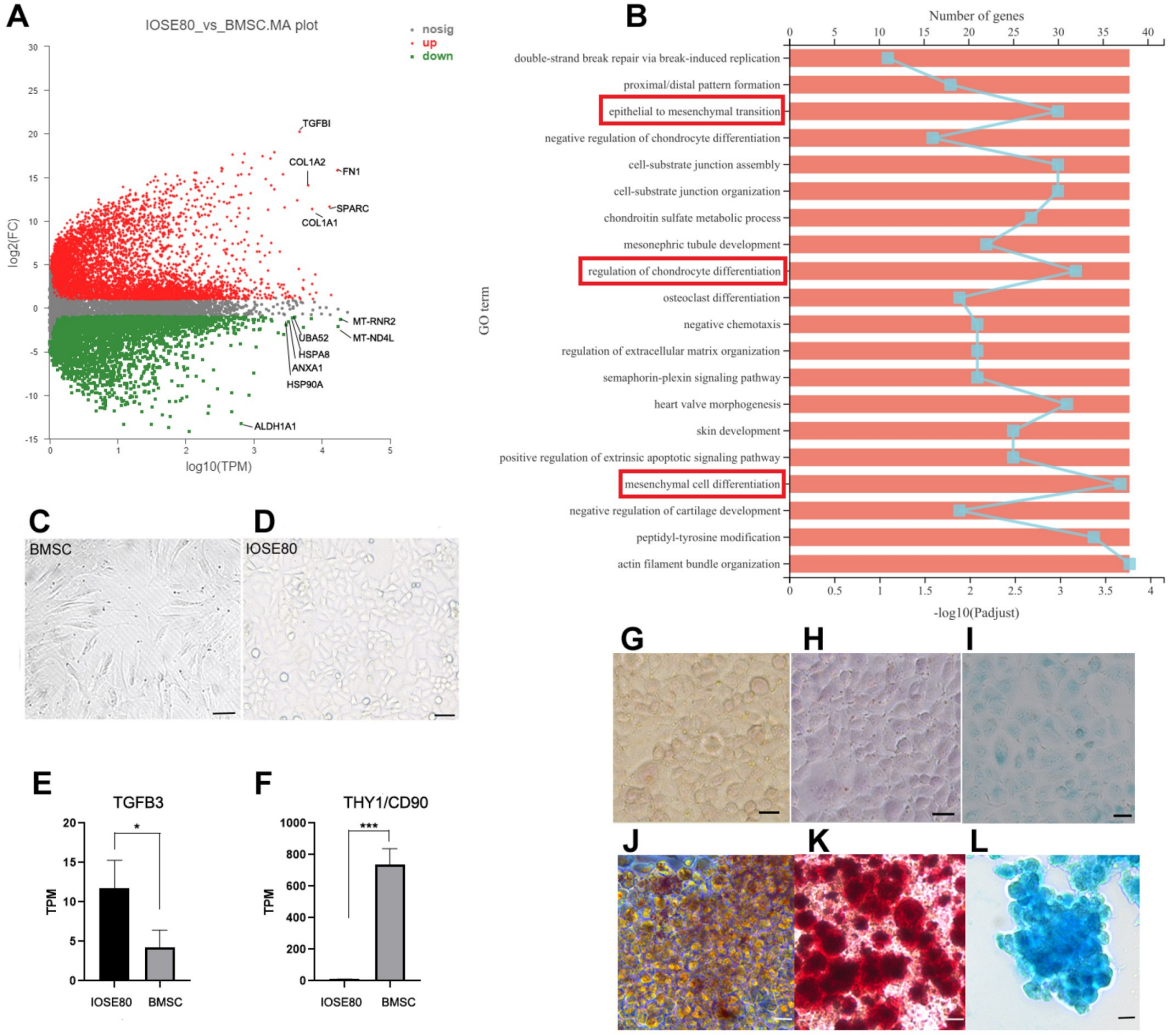
Figure 3 Comparison of transcriptomic differences between IOSE80 cells and BMSCs (A) MA plot of IOSE80 cells vs. BMSCs. Red dots represent upregulated gene expression in MSCs; green dots represent downregulated gene expression in MSCs, in which they are highly expressed in IOSE80 cells. (B) Gene Ontology enrichment analysis of differentially expressed genes shows the top 20 enriched GO terms. The red box indicates the main GO terms of interest. (C) BMSCs show the phenotype of fibroblasts. (D) IOSE80 cells maintain the epithelial cell phenotype under MSC culture conditions. (E-F) Comparative analysis of MSC gene expression in IOSE80 cells and BMSCs. (G-L) IOSE80 cells were cultured under basic culture conditions. Oil red O (G), Alizarin (H), and Alcian (I) were weakly stained as controls. IOSE80 cells were induced into adipocytes as revealed by oil red O staining (J), the induced osteoblasts were stained by Alizarin staining (K), and the chondroblasts were stained by Alcian blue staining (L). BMSCs, bone marrow mesenchymal stem cells; Scale bar: 20 μm.

The transcriptomes of IOSE80 cells and BMSCs were compared. A total of 8928 differentially expressed genes (DEGs) (adjusted *P*<0.05, FC>2) were identified between IOSE80 cells and BMSCs, as shown in Supplementary Table S2. The MA diagram revealed that IOSE80 cells highly expressed mitochondria-related genes, including *MT-RNR2*, *MT-ND4L*, and heat shock family ( *e.g.*, *HSP90A*). On the other hand, BMSCs highly expressed *FN1*, *SPARC*, *COL1A1*, *TGFB1*, *etc*., as depicted in Figure 3A. The differential gene expression analysis also revealed the top 20 enriched GO terms, such as “epithelial to mesenchymal transition”, “regulation of chondrocyte differentiation”, and “mesenchymal cell differentiation” ( Figure 3B). Furthermore, significant enrichment was noted for GO terms, such as “stem cell proliferation”, “stem cell differentiation”, and “wound healing” ( *P*<0.05), as indicated in Supplementary Table S3. In the “stem cell proliferation” category, 18 DEGs were identified, of which 9 genes ( *RUNX1*, *WNT7B*, and *WNT2B*, *etc*.) were found to be highly expressed in BMSCs, while 9 genes ( *NES*/ *Nestin*, *CTC1*, and *WNT3*, *etc*.) were highly expressed in IOSE80 cells ( Supplementary Table S3).

KEGG enrichment analysis showed that “axon guidance”, “focal adhesion”, “ECM receptor interaction”, “cell adhesion molecules”, and “adherens junction” were significantly enriched ( Supplementary Figure S3A and Supplementary Table S4). Reactome pathway analysis indicated that “extracellular matrix organization”, “collage formation”, *etc*., were significantly enriched, suggesting that IOSE80 cells and BMSCs have significant differences in the interaction between cells and extracellular matrix, cell adhesion, and collagen formation ( Supplementary Figure S3B).

The results of the transcriptome analysis revealed that IOSE80 cells had the potential characteristics of mesenchymal stem cells (MSCs). To determine whether IOSE80 cells have the potential to act as MSCs to differentiate into adipocytes, osteoblasts, and chondrocytes, IOSE80 cells were cultured under MSC culture conditions, and IOSE80 cells were passaged for approximately 3‒4 days. The morphology of IOSE80 cells did not change significantly ( Figure 3C,D). The expression histogram showed that IOSE80 cells overexpressed *TGFB3* compared with BMSCs, and BMSCs overexpressed *THY1*/ *CD90* compared with IOSE80 cells ( Figure 3E,F). TGFB3 was noted as a cytokine required for MSCs to induce chondrogenic differentiation [3], suggesting that IOSE80 cells might have the potential to differentiate into chondrocytes *in vitro*.

Then, IOSE80 cells were subjected to induction for differentiation into adipocytes, osteoblasts, and chondrocytes. The induction process was confirmed by oil red O staining, which revealed that numerous cells had undergone adipocyte differentiation. After two weeks of induction, lipid droplets were observed in the initial IOSE80 cells. Similarly, after one month of differentiation, Alizarin staining indicated that the cells were differentiated into osteoblasts, while Alcian blue staining confirmed the differentiation of the cells into chondrocytes ( Figure 3G‒L). These findings suggested that IOSE80 cells possess the potential to function as MSCs and can differentiate into adipocytes, osteoblasts, and chondroblasts *in vitro*.

### The IOSE80 cell line has the characteristics of PSCs

**Figure 4:**
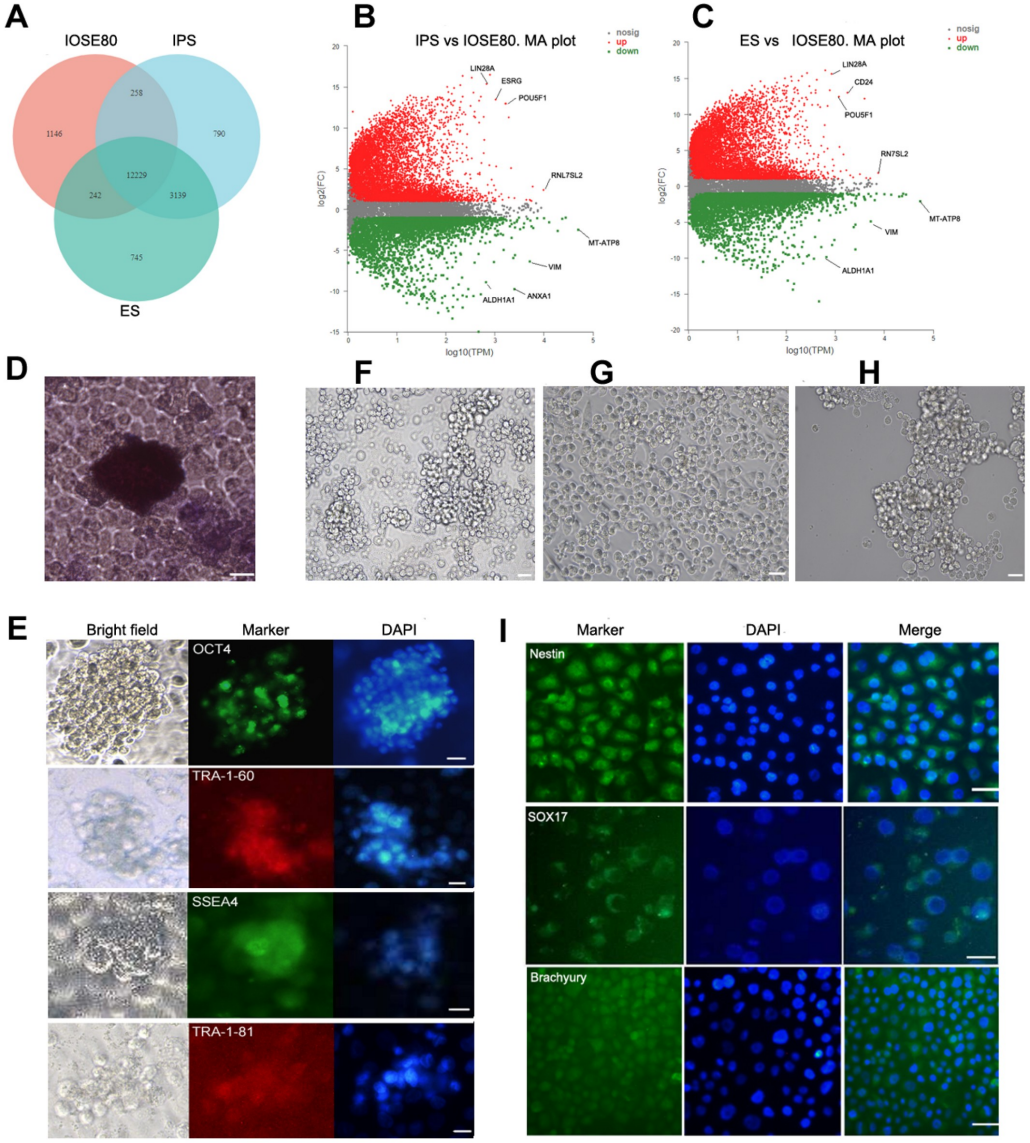
Figure 4 Comparison of transcriptomic differences between IOSE80 cells and pluripotent stem cells (A) Venn diagram analysis shows that IOSE80 cells, IPS, and ES share 12,229 genes. (B) MA plot of IPS vs. IOSE80 cells. Red dots represent upregulated gene expression in IPS cells, and green dots represent downregulated gene expression in IPS cells, in which they are highly expressed in IOSE80 cells. (C) MA plot of ES vs. IOSE80 cells. Red dots represent upregulated gene expression in ES cells; green dots represent downregulated gene expression in ES cells, in which they are highly expressed in IOSE80 cells. (D) IOSE80 cells form colonies in stem cell culture, showing positive alkaline phosphatase staining. (E) IOSE80 cell colonies express pluripotency markers, including OCT-4, SSEA4, TRA-1-60, and TRA-1-81. IOSE80 cells were induced to differentiate into ectoderm (F), mesoderm (G), and endoderm (H) in vitro for one week. (I) Immunofluorescence staining shows that IOSE80 cells express markers for all three germ layers, namely, Nestin (ectoderm), Brachyury (mesoderm), and SOX17 (endoderm), indicating their differentiation. IPS, induced pluripotent stem cells. ES cells, embryonic stem cells. DAPI, 4′,6-diamidino-2-phenylindole. Scale bar: 20 μm.

To demonstrate the characteristics of PSCs, IOSE80 cells were compared with PSCs (IPS cells and ES cells, respectively) in the transcriptome. Venn diagram analysis showed that IOSE80 cells, ES cells, and IPS cells shared 12,229 genes ( Figure 4A). The MA map revealed that *MT-ATP8*, *VIM*, *ANXA1*, and *ALDH1A1*, *etc*., were overexpressed in IOSE80 cells; *POU5F1*, *ESRG*, *LIN28A*, etc., were overexpressed in IPS cells, while *POU5F1*, *CD24*, *LIN28A*, *etc*., were overexpressed in ESCs ( Figure 4B,C). As shown in the previous PCA, ES and IPS cell transcription profiles were similar. These two groups of data were integrated as PSCs. Comparison of IOSE80 cells with PSCs indicated that 10,077 differential transcripts were obtained ( Supplementary Table S5).

The GO enrichment analysis showed that there was significant enrichment in “wound healing”, “regulation of stem cell proliferation”, “response to estradiol”, “epithelial cell proliferation”, “tight junction”, and “collagen binding” ( *P*<0.05) ( Supplementary Table S6). There were 26 DEGs in the GO term “regulation of stem cell proliferation”. Among them, 10 genes ( *TGFB1*, *TBX3*, *SNAI2*, *SMARCD3*, *CITED1*, *VAX1*, *LTBP3*, *NF2*, *ZNF335*, and *SOX18*) were overexpressed in IOSE80 cells, and 16 genes ( *KITLG*, *NANOG*, *EPCAM*, *etc*.) were highly expressed in PSCs. There were 35 DEGs in the GO term “epithelial cell proliferation”, of which the highly expressed genes in IOSE80 cells included *LGR5*, *HGF*, and *SERPINB1*, whereas the highly expressed genes in PSCs included *BMP4*, *CCN3*, and *TNFSF11*.

The KEGG pathway analysis showed that signaling pathways, such as “calcium signaling pathway”, “ECM-receptor interaction”, “MAPK signaling pathway”, “cAMP signaling pathway”, and “signaling pathways regulating pluripotency of stem cells”, were significantly enriched ( *P*<0.05; Supplementary Table S7). There were 78 DEGs in “signaling pathways regulating pluripotency of stem cells”, of which *SOX2*, *KLF4*, *NANOG*, and *FGF2* were highly expressed in PSCs, whereas *PAX6*, *JAK2*, *LIF*, and *OTX1* were highly expressed in IOSE80 cells.

To further demonstrate whether IOSE80 cells have the characteristics of pluripotent stem cells, IOSE80 cells were cultured in TeSR ^TM^ for 3 days, and they were then transferred onto the feeder of hAECs for 3‒4 days according to our previous research [29]. The results showed that IOSE80 cells could form embryonic stem cell-like colonies on the feeder of hAECs, and these colonies exhibited alkaline phosphatase-positive staining ( Figure 4D), expressing PSC markers, including Oct4, SSEA4, TRA-1-60, and TRA-1-81 ( Figure 4E). We then attempted to clarify whether IOSE80 cells had the ability to differentiate into three germ layers *in vitro*. After one week of culture under the tri-lineage differentiation culture conditions, the cell morphology changed ( Figure 4F‒H), and immunostaining showed the expressions of the ectoderm marker Nestin, mesoderm marker SOX17, and endoderm marker Brachyury, suggesting that IOSE80 cells have the potential to differentiate into PSCs ( Figure 4I).

### The IOSE80 cell line has the characteristics of germ cells

**Figure 5:**
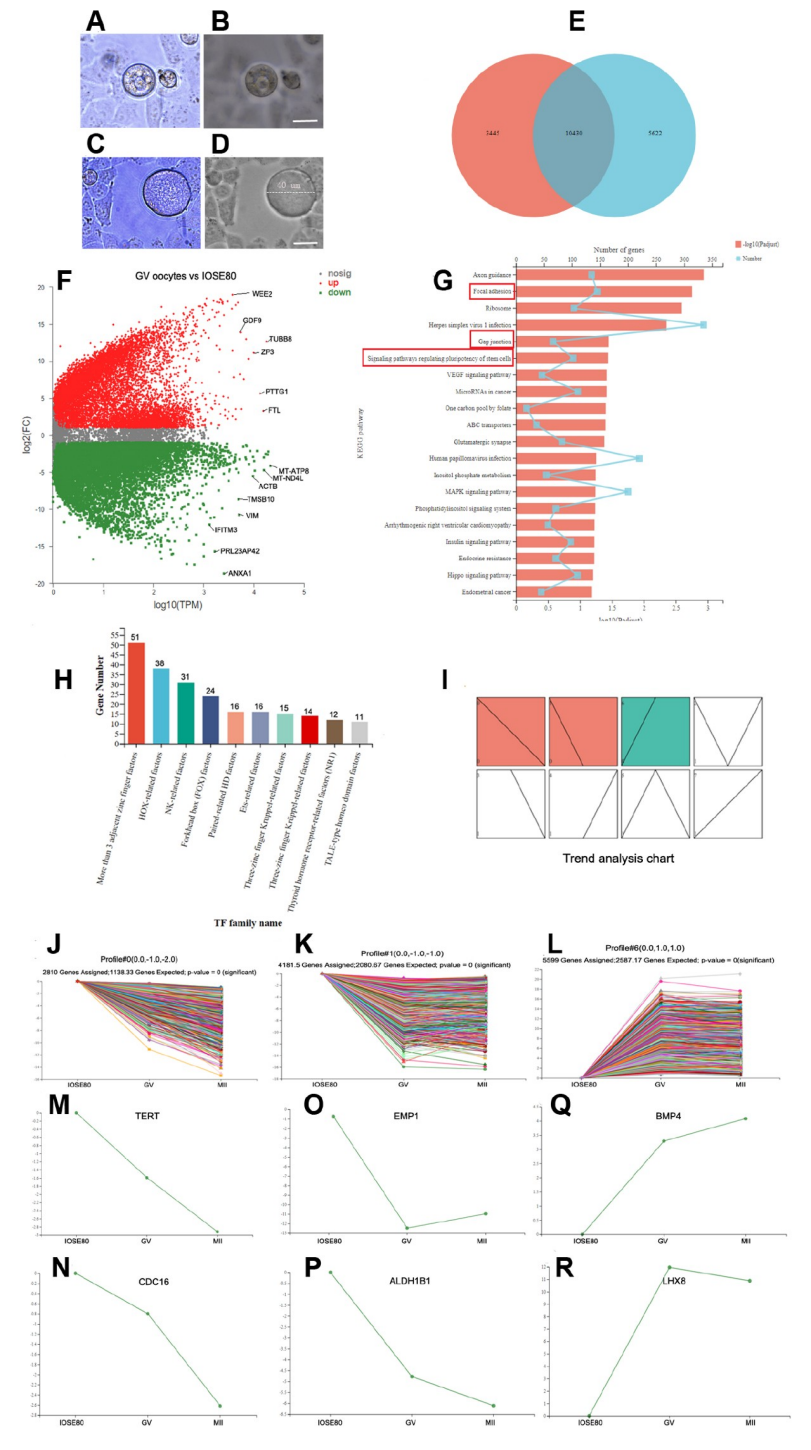
Figure 5 Comparison of transcriptomic differences between IOSE80 cells and germ cells (A‒D) IOSE80 cells spontaneously differentiate into oocyte-like cells in vitro under a phase-contrast microscope. Scale bar: 20 μm. (E) Venn diagram analysis. Venn diagram analysis shows that IOSE80 cells and GV oocytes share 16,596 genes. (F) MA plot of GV oocytes vs. IOSE80 cells. Red dots represent upregulated genes in GV oocytes, and green dots represent downregulated genes in GV oocytes, in which they are highly expressed in IOSE80 cells. (G) KEGG pathway analysis shows that signaling pathways, such as “focal adhesion”, “gap junction”, and “signaling pathways regulating pluripotency of stem cells”, are enriched. (H) Statistical analysis reveals shared transcription factor families between IOSE80 cells and GV oocytes. (I) Time series expression trend analysis of IOSE80 cells and oocytes at the GV and MII stages. Colored modules represent differences in gene expression. Red modules represent the downward trend of expression (P<0.05). The green module represents the upward trend of expression (P<0.05). (J,K) The trend of expression levels of all genes in the red module (Profile # 0 J and Profile # 1 K). (L) The trend of expression levels of all genes in the green module (Profile # 6). (M,N) Representative genes TERT and CDC16 in red module profile # 0. (O,P) Representative genes EMP1 and ALDH1B1 in red module Profile # 1. (Q,R) Representative genes BMP4 and LHX8 in the blue module Profile # 6.

It was previously reported that epithelial cells derived from human follicular fluid could spontaneously differentiate into oocyte-like structures *in vitro* [29], and we attempted to clarify whether IOSE80 cells have the potential to spontaneously differentiate into oocytes. After 4 days of culture, some IOSE80 cells were enlarged and spontaneously differentiated into round-shaped oocyte-like structures attached to the underlying cells ( Figure 5A,B). Notably, the size of round-shaped oocyte-like cells could further enlarge to a diameter of 40 μm ( Figure 5C,D).

The transcriptomic difference between IOSE80 cells and germ cells was further analyzed. After comparison of IOSE80 cells with oocytes at the GV stage, a total of 15,682 differential transcripts were obtained ( Supplementary Table S8). Venn diagram showed that IOSE80 cells and GV oocytes shared 10,430 genes ( Figure 5E).

The MA plot of GV oocytes and IOSE80 cells indicated that GV oocytes highly expressed *ZP3*, *GDF9*, *FTL*, *WEE2*, and *PTTG1*, while IOSE80 cells highly expressed *MT-ATP8*, *ACTB*, *VIM*, *IFITM3*, and *ANXA1* ( Figure 5F). The GO enrichment analysis between GV oocytes and IOSE80 cells revealed that “positive regulation of canonical Wnt signaling pathway”, “noncanonical Wnt signaling pathway”, “glycoprotein metabolic”, and “response to estradiol” were significantly enriched ( Supplementary Table S9), suggesting that Wnt signaling and estrogen play an important role in the differentiation process of IOSE80 cells into GV oocytes. GSEA indicated two gene sets related to germ cell development. The GOBP “germ_cell_development” (ES: 0.350, NES: 2.375, Padjust: 0.002) was identified to have a leading edge consisting of 91 genes. In GV oocytes, high expression levels were observed in genes, such as *WEE2*, *KIT*, *SOHLH2*, and *NOBOX*, while *LIN28A*, *MLH1*, and *CCNB1IP1* exhibited higher expression levels in IOSE80 cells ( Supplementary Figure S4A). Similarly, the GOBP “meiotic cell cycle process” (ES: 0.413, NES: 2.593, Adjust: 0) showed a leading edge comprising 89 genes. High expression levels of genes, such as *WEE2*, *OOEP*, and *SYCP3*, were noted in GV oocytes, whereas higher expression levels of *MLH1*, *MSX1*, and *BTBD18* were identified in IOSE80 cells ( Supplementary Figure S4B). The KEGG pathway analysis revealed that signaling pathways, including “focal adhesion”, “gap junction”, and “signaling pathways regulating pluripotency of stem cells”, were enriched ( Figure 5G and Supplementary Table S10).

The transcription factor family plays an important role in oocyte derivation. Various types of transcription factors were analyzed and counted ( Figure 5H). More than 3 adjacent zinc finger factors contained most transcription factors, accounting for a total of 51 transcription factors, mainly including *SNAI1*, *YY1*, *CTCF*, *ZNF263*, and so on. Secondly, HOX-related factors included 38 transcription factors, mainly involving *HOXA1*, *MEOX1*, *MSX1*, etc. In addition, the forkhead box (FOX) family of transcription factors comprised 24 factors, with key members, such as FOXO3, FOXA1, and FOXL1. To guide the transcription of IOSE80 cells to oocytes *in vitro*, the STEM algorithm was employed to analyze the transcription change trend from IOSE80 cells to GV oocytes and then to MII oocytes. A total of 8 modules were obtained, of which 3 modules exhibited significant differences (profile# 0, profile# 1, profile# 6) ( Figure 5I and Supplementary Table S11). In the profile# 0 module, a decrease in telomerase reverse transcriptase ( *TERT*), cell division cycle-related genes ( *CDC16* and *CDC34*), epithelial cell rupture-related gene ( *PLAUR*), and TGFβ signaling pathway-related gene ( *TGFB3*) was found in IOSE80 cells, as well as in GV stage oocytes to MII stage oocytes ( Figure 5J,M,N and Supplementary Figure S5A‒C). In the profile 1 module, a downwards trend was noted in the expression levels of epithelial membrane proteins (EMP1 and EMP3), epithelial cell cytoskeleton (KRT10), epithelial stem cell marker ALDH1B1, and epithelial cell growth and endocrine regulatory factor HGF( Figure 5K,O,P and Supplementary Figure S5D‒F). In the profile# 6 module, an upwards trend was observed in the expression levels of cell transcription factors LHX8, STAT3, retinoic acid response gene ( *STRA8*), meiotic gene ( *SCP3*), cell growth factors (EGF and BMP4), and germ cell markers (DAZL and GDF9) ( Figure 5L,Q,R, and Supplementary Figure S5G‒L).

**Figure 6:**
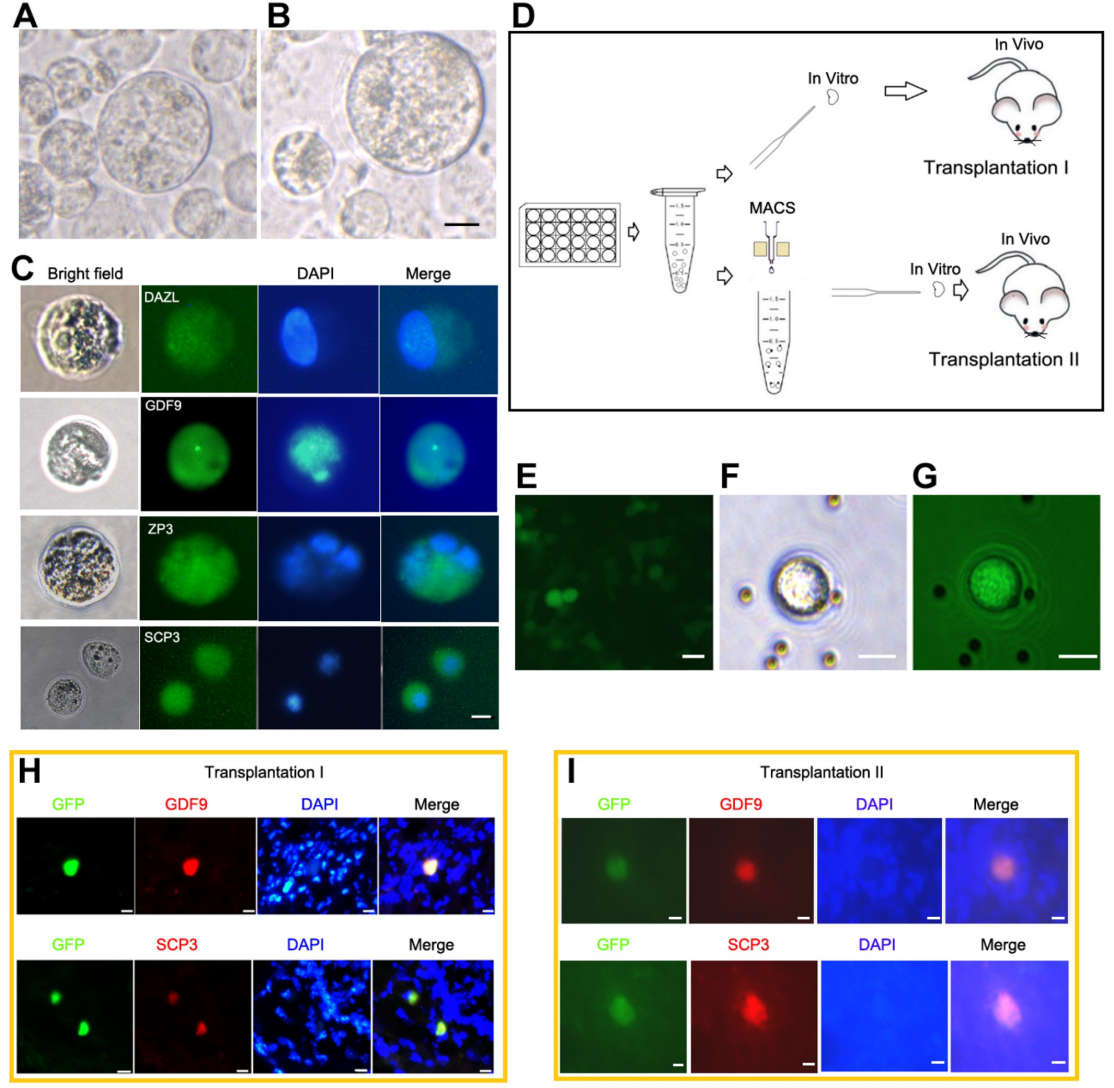
Figure 6 IOSE80 cells could differentiate into germ cells *in vitro* and *in vivo* (A,B) IOSE80 cells are induced to differentiate under the conditions of retinoic acid, cytokines, and hormones. Oocyte-like cells on day 15 (A). Oocyte-like cells on day 25 (B). (C) The induced cells express germ cell-related markers, including DAZL, GDF9, ZP3, and SCP3, as evidenced by immunofluorescence. (D) Schematic diagram of germ cell differentiation via in vitro transplantation. In the transplantation I protocol, IOSE80 cells (50 μL, 1.3×105 cells) are transplanted into mouse ovaries in vitro, and then the ovaries loaded with cells are transplanted subcutaneously into SCID-NOD mice (8 weeks old, female). The transplantation II scheme, in which IOSE80 cells are first sorted by IFITM3 (fragilis) magnetic beads and then transplanted as protocol I. MACS, Magnetic activated cell sorting. (E) IOSE80 cells are transfected with GFP lentivirus. (F) IFITM3-positive IOSE80 cells are sorted. (G) Fluorescence shows that IFITM3-positive IOSE80 cells carried GFP green fluorescence. (H) The transplantation I scheme represents GFP-positive cells expressing GDF9 or SCP3. (I) The Transplantation II scheme represents GFP-positive IOSE80 cells expressing GDF9 or SCP3 after IFITM3 antibody-positive sorting transplantation. Scale bar: 20 μm.

According to the trend analysis and combined with other germ cell derivation methods [ 23‒ 29], the *in vitro* oocyte derivation scheme of IOSE80 cells was optimized. IOSE80 cells were induced into oocyte-like cells under hanging drop conditions and sequential culture with the addition of retinoic acid, cytokines, and hormones. On day 15 of differentiation, IOSE80 cells were induced into oocyte-like cells ( Figure 6A). On day 25 of differentiation, the cell diameter was further enlarged to 60-70 μm ( Figure 6B), and the cells expressed oocyte markers, including DAZL, SCP3, GDF9, and ZP3 ( Figure 6C).

To further study whether IOSE80 cells could differentiate into germ cells *in vivo*, a transplantation mouse model was established ( Figure 6D). Lentivirus packaged with GFP was transfected into IOSE80 cells, followed by transplantation of the transfected cells into a mouse ovary. Subsequently, the ovary was xenografted subcutaneously into NOD-SCID mice. To address the possible heterogeneity of ISOE80 cell lines, IFITM3 ^+^/GFP ^+^ cells were sorted from ISOE80 cells using an antiIFITM3 antibody. The sorted cells were then transplanted into a mouse ovary, and the ovary was further xenografted subcutaneously into NOD-SCID mice ( Figure 6E‒G). Two weeks after transplantation, immunofluorescence analysis revealed the expression levels of the germ cell markers SCP3 and GDF9 in both unselected and IFITM3 ^+^-sorted GFP-positive cells ( Figure 6H,I).

These results suggested that IOSE80 cells might have the potential to differentiate into oocyte-like cells.

### IOSE80 cells may be involved in ovarian repair function *in vivo*

**Figure 7:**
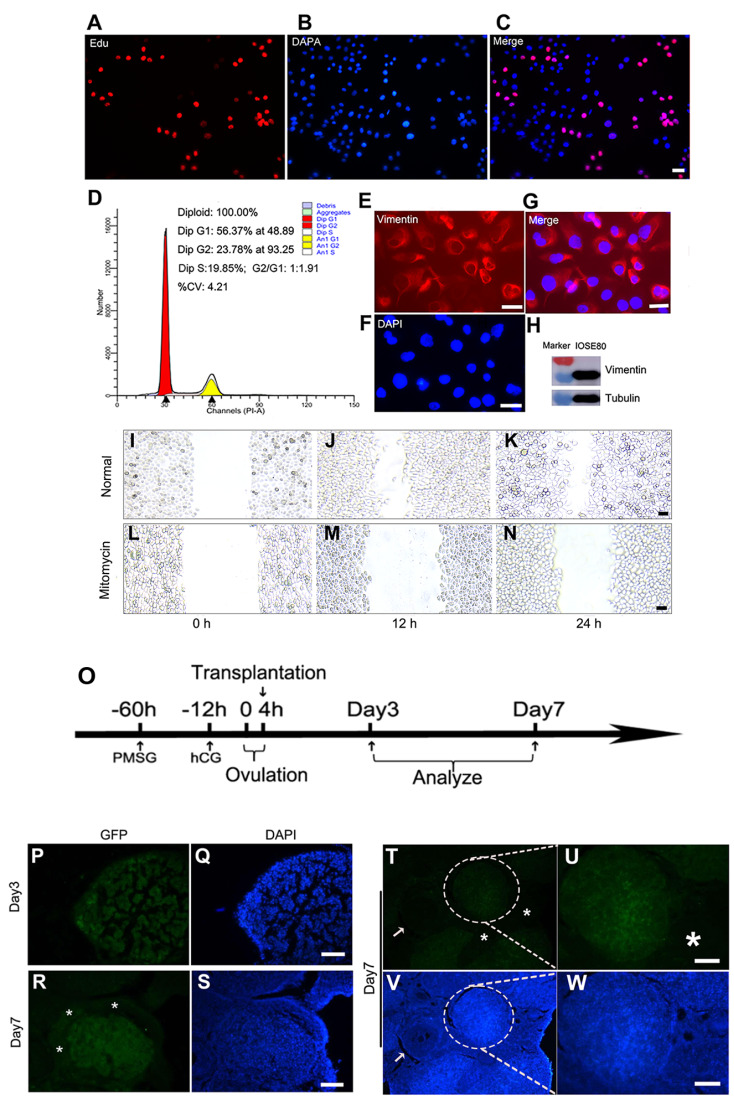
Figure 7 Transplantation of IOSE80 cells for ovarian repair in superovulated mice (A‒C) The proliferation ability of IOSE80 cells was assessed by EdU staining. Red is indicative of EdU-stained cells, blue is indicative of Hoechst 33342-stained cells, and purplish red represents proliferating cells after merging. (D) Flow cytometry analysis showed that 56.37% of IOSE80 cells were in G1 phase, 23.78% were in G2 phase, and 19.85% were in S phase. (E‒G) Immunofluorescence showed that Vimentin was highly expressed in the cytoplasm. (H) Western blot analysis indicated the expression level of Vimentin in IOSE80 cells. (I‒N) Wound healing assay of IOSE80 cells. IOSE80 cells migrate at 0 h, 12 h and 24 h in RPMI-1640 medium (I-K). IOSE80 cells show migration inhibition at the corresponding time after mitomycin treatment (L‒N). (O) Flowchart of superovulation and cell transplantation in vivo. Eight-week-old ICR mice (n=20) received an intraperitoneal injection of 5 IU pregnant mare serum gonadotropin (PMSG) (‒60 h), followed by 5 IU human chorionic gonadotropin (hCG) at 44-46 h after PMSG injection (‒12 h). Twelve hours after hCG injection, the mice began to ovulate (0 h), and most of the mice completed ovulation approximately 4 h later. IOSE80 cells were collected and transplanted into the ovaries of mice (50 μL, 5×105 cells) 16 h after the injection of hCG. The murine ovaries were collected on days 3 and 7 after cell transplantation. (P-Q) The follicles of superovulated recipient mice on day 3 after cell transplantation showed GFP-positive cells stained with DAPI. (R,S) On day 7 after cell transplantation, the follicles of superovulated recipient mice showed GFP-positive cells (R) with DAPI staining (S). *The asterisk represents the murine GFP-negative ovarian cells. (T,W) On day 7 after cell transplantation, the follicles of superovulated recipient mice showed transplanted GFP-positive cells (T) and magnified vision (U), referring to DAPI staining (V,W). The white arrow indicates the murine recipient oocyte (GFP-negative) (T) and DAPI staining (V). *The asterisk represents the murine GFP-negative ovarian cells. Scale bar: 20 μm.

The proliferation and migration of OSE cells play an important role in the repair of the ruptured ovarian surface after ovulation. This recurring process suggests that the ruptured wound is replenished by stem/progenitor cells in OSE cells [ 23‒ 28]. To investigate the proliferation, migration, and repair functions of IOSE80 cells, the proliferation ability of IOSE80 cells was assessed by EdU staining, and the results showed that IOSE80 cells were proliferating ( Figure 7A‒C). Next, the proportion of IOSE80 cells in different cell cycles was analyzed, and the effect of SV-40 transfection on chromosome ploidy was assessed. Flow cytometry showed that 56.37% of IOSE80 cells were in G1 phase, 23.78% were in G2 phase, and 19% of IOSE80 cells were in S phase (more than 15%), suggesting that IOSE80 cells had a strong proliferation ability *in vitro* ( Figure 7D). In addition, aneuploid cells were not detected after long-term culture *in vitro*, indicating that SV40T transfection did not affect chromosome ploidy of IOSE80 cells.

Previous studies showed that primary culture of human ovarian epithelial cells lost the polarity of cells and several markers of epithelial cells but obtained the marker of mesenchymal-like cells during cell subculture [1]. Immunofluorescence staining showed that Vimentin, an EMT-related marker, was located in the cytoplasm of cells ( Figure 7E‒G). Western blot analysis indicated high expression of Vimentin in IOSE80 cells ( Figure 7H), and EMT was involved in the repair process of ovarian epithelial cells after ovulation [7]. According to the GO enrichment analysis of “epithelial to mesenchymal transition” in IOSE80 cells and BMSCs, there were 29 DEGs in “epithelial to mesenchymal transition”, of which 11 genes (including *HEY1*, *FAM83D*, and *SPRY1*, *etc*.) were highly expressed in IOSE80 cells, while 18 genes (including *SNAI2*, *SNAI1L*, *ZEB1*, *TWIST*, *etc*.) were highly expressed in BMSCs ( Supplementary Figure S6A‒F). In addition, the EMT-related transcription factors *ZEB1* and *TWIST* were expressed in IOSE80 cells, although they were not highly expressed in BMSCs. In combination with the high expression levels of Vimentin and these EMT regulatory genes in IOSE80 cells, it was suggested that partial EMT (pEMT) could occur in the culture of IOSE80 cells *in vitro*.

Then, the *in vitro* wound healing properties of IOSE80 cells were evaluated. Confluence was achieved by culturing the cells, in which wounds were created on the cell layers using sterile pipette tips. Subsequently, the cells were incubated for 0, 12, and 24 h in the presence or absence of mitomycin C (10 μg/mL). A significant decrease in IOSE80 cell migration was observed with mitomycin C treatment, suggesting that cell division and migration were necessary for *in vitro* wound healing of IOSE80 cells ( Figure 7I‒N).

Comparison of the transcriptome of IOSE80 cells with that of BMSCs and PSCs for different genes in GO terms related to “wound healing” showed that there were 35 DEGs in “wound healing”, of which 8 genes ( *TGFB3*, *NLRP6*, *FUT10*, *SCARB1*, *ERBB3*, *CELSR1*, *ALOX15P1*, and *EPPK1*) were highly expressed in IOSE80 cells, while 26 genes ( *TGFB1*, *COL3A1*, *FN1*, *etc*.) were highly expressed in BMSCs ( Supplementary Table S2). There were 36 DEGs in “wound healing”, of which 10 genes ( *TGFB1*, *TGFB3*, *ELK3*, *DCN*, *SLC11A1*, *SCARB1*, *EREG*, *COL3A1*, *MAP3K5*, and *C6orf89*) were highly expressed in IOSE80 cells, while 26 genes ( *DSP*, *CCM2L*, *WNT5B*, *etc*.) were highly expressed in PSCs ( Supplementary Table S2). Therefore, these findings indicated that IOSE80 cells exhibited a distinct process in wound healing compared to BMSCs and PSCs.

We further studied the ovarian repair function of IOSE80 cells, and a superovulation mouse model was established. In brief, after ovulation induction, GFP-transfected IOSE80 cells were transplanted into ovarian cysts, and the kinetics of transplantation were observed after transplantation ( Figure 7O). Immunofluorescence showed that GFP-positive IOSE80 cells migrated into the postovulatory cavity and participated in ovarian remodeling. After 3 days of superovulation, GFP-positive cells were relatively loose ( Figure 7P,Q). After 7 days of superovulation, GFP-positive cells became denser, while the murine ovarian cells were GFP-negative, forming a chimeric structure ( Figure 7R,S). To further demonstrate whether GFP-positive IOSE80 cells could migrate into several postovulation follicular cavities, another murine ovarian section on day 7 after transplantation was used for immunofluorescence analysis. Referring to DAPI staining ( Figure 7V,W), it was noted that GFP-negative cells outside the follicle and oocytes of recipient mice and the transplanted GFP-positive cells formed chimeric structures ( Figure 7T,U), suggesting that IOSE80 cells could migrate, locate, and be involved in the superovulation repair function *in vivo*.

## Discussion

In this study, we compared IOSE80 cells with MSCs, pluripotent stem cells, and germ cells by high-throughput sequencing and revealed that IOSE80 cells have the characteristics of MSCs, pluripotent stem cells, and germ cells, which are similar to the characteristics of epithelial cells derived from human follicular fluid. In addition, IOSE80 cells are multipotent epithelial cells with robust proliferation, migration, and repair functions.

It has been previously demonstrated that human ovarian surface epithelial cells are multipotent [ 16, 23‒ 29]. In the present study, we, for the first time, demonstrated this through transcriptomics analysis. By comparing IOSE80 cells with BMSCs, we found 18 DEGs in “stem cell proliferation”, of which 9 genes were highly expressed in BMSCs, and 9 genes ( *CTC1*, and *NES*, *etc*.) were highly expressed in IOSE80 cells. The trimeric CST complex is composed of CTC1, STN1 (oligonucleotide/oligosaccharide-binding fold-containing protein 1), and TEN1 (telomerase capping complex subunit homolog), which are involved in telomere maintenance and genome stability [32]. CTC1 deletion leads to telomere replication defects, telomere loss, and stem cell exhaustion [33]. The expression of Nestin was reported to be upregulated in most mitotic cells and downregulated in all cells upon differentiation [34]. Méndez-Ferrer *et al*. [35] reported the differentiation of *Nes*-GFP ^+^ cells into mesenchymal stem cell lineages under adherent culture conditions, as evidenced by the progressive upregulation of osteoblastic, adipocytic, and chondrocytic differentiation genes, ultimately leading to the acquisition of a mature phenotype after one month. Additionally, Nestin ^+^ cells exhibited multipotency and self-renewal capacity by forming mesenspheres. Compared with BMSCs, a high expression level of Nestin in IOSE80 cells was identified, which might be related to their robust mitosis. Compared with PSCs, we identified 25 DEGs in the GO term of “regulation of stem cell proliferation”, of which 10 genes ( *TGFB1*, *SMARCD3*, *TBX3*, *etc*.) were overexpressed in IOSE80 cells. *Smarcd3*/ *Baf60c* expression in immortalized human breast epithelial cells upregulated the expression of *Wnt5a* to promote epithelial mesenchymal transition [36]. Compared with PSCs, the highly expressed *SMARCD3* in IOSE80 cells regulated stem cell proliferation, which might endow IOSE80 cells with the characteristics of MSCs and promote pEMT. In the GO term of “epithelial cell proliferation”, *LGR5* and *HGF* were highly expressed in IOSE80 cells compared with PSCs. It has been reported that hepatocyte growth factor (HGF), keratinocyte growth factor, and kit ligand have autocrine interactions in the regulation of normal ovarian epithelial cells [37]. Therefore, the transcriptome results provide further confirmation that IOSE80 cells include a set of stem cell genes distinct from BMSCs and PSCs, playing an important role in regulating the proliferation of ovarian epithelial stem cells.

It has been widely reported that oocyte-like cells can be spontaneously derived from ovarian epithelial stem cells, as evidenced in previous studies [ 23‒ 28] and our previous research [29]. Oocyte-like structures can also be generated through the EB pathway using embryonic stem cells [ 38, 39]. In the present study, embryoid bodies were found to be formed from IOSE80 cells induced by hanging drops, in the presence of bFGF, EGF, and other growth factors, along with murine ovarian granulosa cells as feeder cells to mimic the ovarian microenvironment. Subsequently, RA, BMP4, and hormones were added to further induce IOSE80 cells. The time series expression trend analysis revealed that *BMP4* expression was upregulated from IOSE80 cells to MII oocytes, and BMP4 was also added at the late stage when oocyte-like cells were derived from IOSE80 cells. Transcriptome analysis demonstrated that IOSE80 cells expressed PPT8 genes ( *LHX8*, *STAT3*, and *SOHLH1*), as well as eight transcription factor genes essential for the transition from primordial to primary follicles [40], suggesting that these genes might constitute the critical molecular basis for oocyte-like cells to be derived from IOSE80 cells.

The inflammatory process characterizes ovarian ovulation. The association among ovarian epithelium, basal cells, and the polarity of the original epithelial cells is destroyed before ovarian ovulation, as reported previously [ 1, 7]. Subsequently, TGFB1 and inflammatory factors present in the follicular fluid can impact OSE through the basal layer, resulting in the epithelial to mesenchymal transition of cells [15]. Studies have demonstrated that ovarian epithelial cells lose some epithelial cell characteristics and express mesenchymal cell markers, such as Vimentin [ 1‒ 4], with an increase in culture time *in vitro*. The findings of the present study indicated that IOSE80 cells expressed EMT-related markers, including SNAI1, SNAI2, ZEB1, ZEB2, and TWIST1, suggesting that these cells experienced pEMT *in vitro*. Cell migration was observed along with the proliferation of IOSE80 cells during the wound healing assay. Therefore, IOSE80 cells exhibited the characteristics of proliferation and migration, which would be similar to the features of ovarian epithelial cells around follicles *in situ* before ovulation. Moreover, further *in vivo* results showed that IOSE80 cells displayed robust migration dynamics and self-organization ability. Immunofluorescence revealed that GFP-carrying IOSE80 cells formed a corpus luteum-like structure on day 7 after transplantation, which resembled a “patch” after wound healing [ 41, 42].

In conclusion, we demonstrated that the stem cell line derived from the ovarian surface epithelium, specifically immortalized IOSE80 cells could be utilized for further investigation of their potential regenerative role. This includes their ability to potentially undergo oogenesis *in vitro* and differentiate into other types of somatic cells, thereby making them an appropriate candidate for future tissue engineering and regenerative medicine.

## Supporting information

23354Table_S1

23354Table_S4

23354Table_S10

23354Table_S9

23354Table_S3

23354Table_S7

23354Table_S8

23354Table_S5

23354Table_S11

23354Table_S2

23354Table_S6

23354Supplementary_Figure

## Supplementary Data

Supplementary data is available at *Acta Biochimica et Biophysica Sinica* online.
