# Supplementary material for: Identification and characterization of multipotential stem cells in immortalized normal ovarian surface epithelial cells: The pluripotency of immortalized normal ovarian surface epithelial cells
Source: Acta Biochim Biophys Sin (Shanghai). 2024 Jan 19;56(2):239–54. doi: 10.3724/abbs.2023253 (PMC10984850; doi:10.3724/abbs.2023253)
Supplement: 23354Supplementary_Figure [file 23354Supplementary_Figure.docx]

**
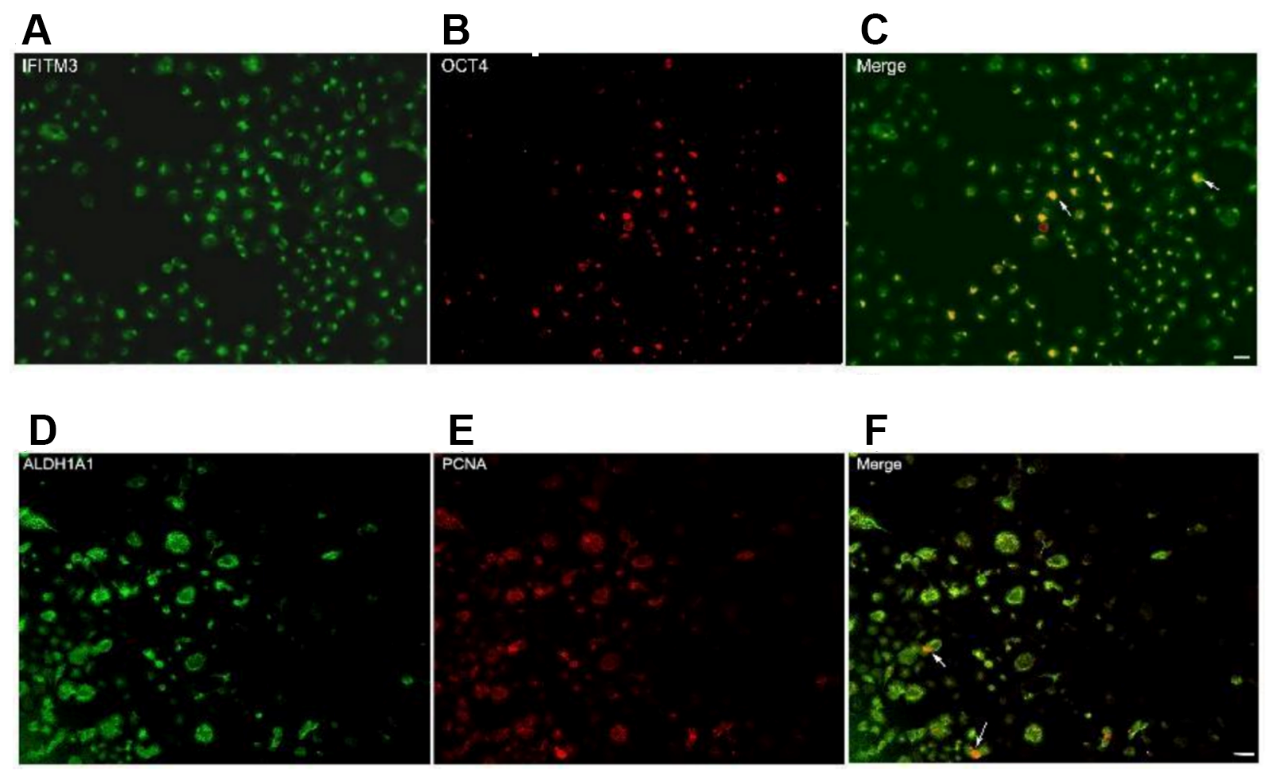
Supplementary Figure S1. Immunofluorescence staining for IOSE80 cell proliferation and stem cell markers** (A) Cytoplasmic IFITM3 staining, (B) nuclear OCT4 staining, and (C) merge of IOSE80 cells. Arrows indicate double-labelled cells. (D) Cytoplasmic ALDH1A1 staining, (E) nuclear PCNA staining, and (F) merge of IOSE80 cells. Arrows indicate double-labelled cells.


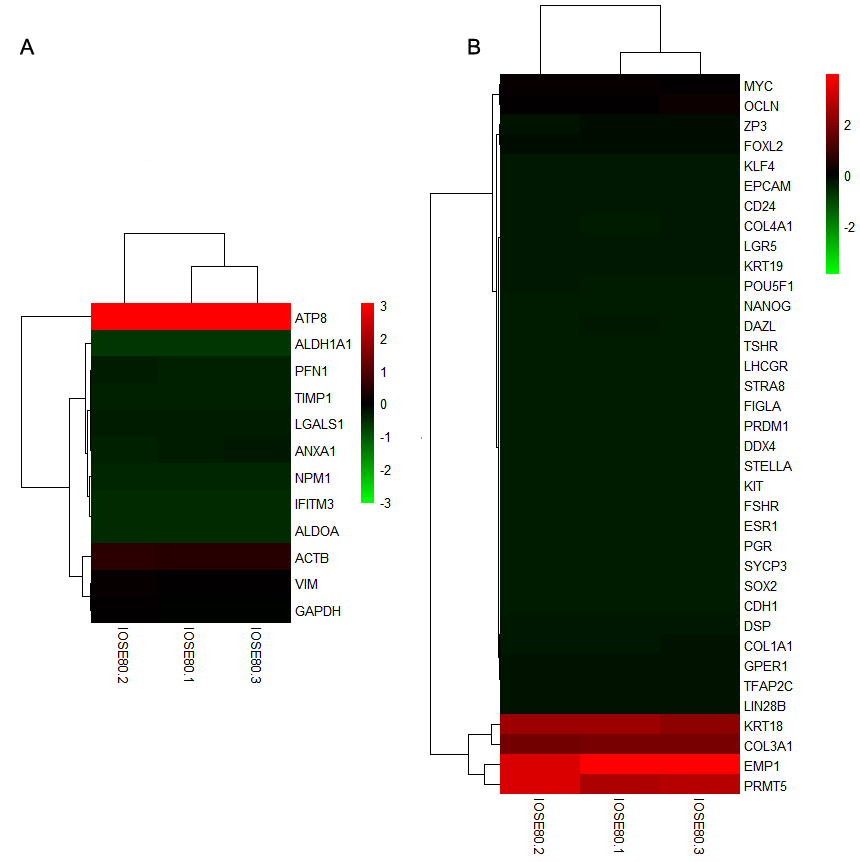


**Supplementary Figure S2. Heatmap of IOSE80 cell gene expression** (A) Clustering and expression levels of highly expressed genes in IOSE80 cells. The expression of the *MT-ATP8* gene was the highest in IOSE80 cells. (B) Clustering and expression of germline, stem cell and epithelial cell-related marker genes in IOSE80 cells. *EMP1* has the highest expression level in this set of genes and has the closest cluster with *PRMT5*.


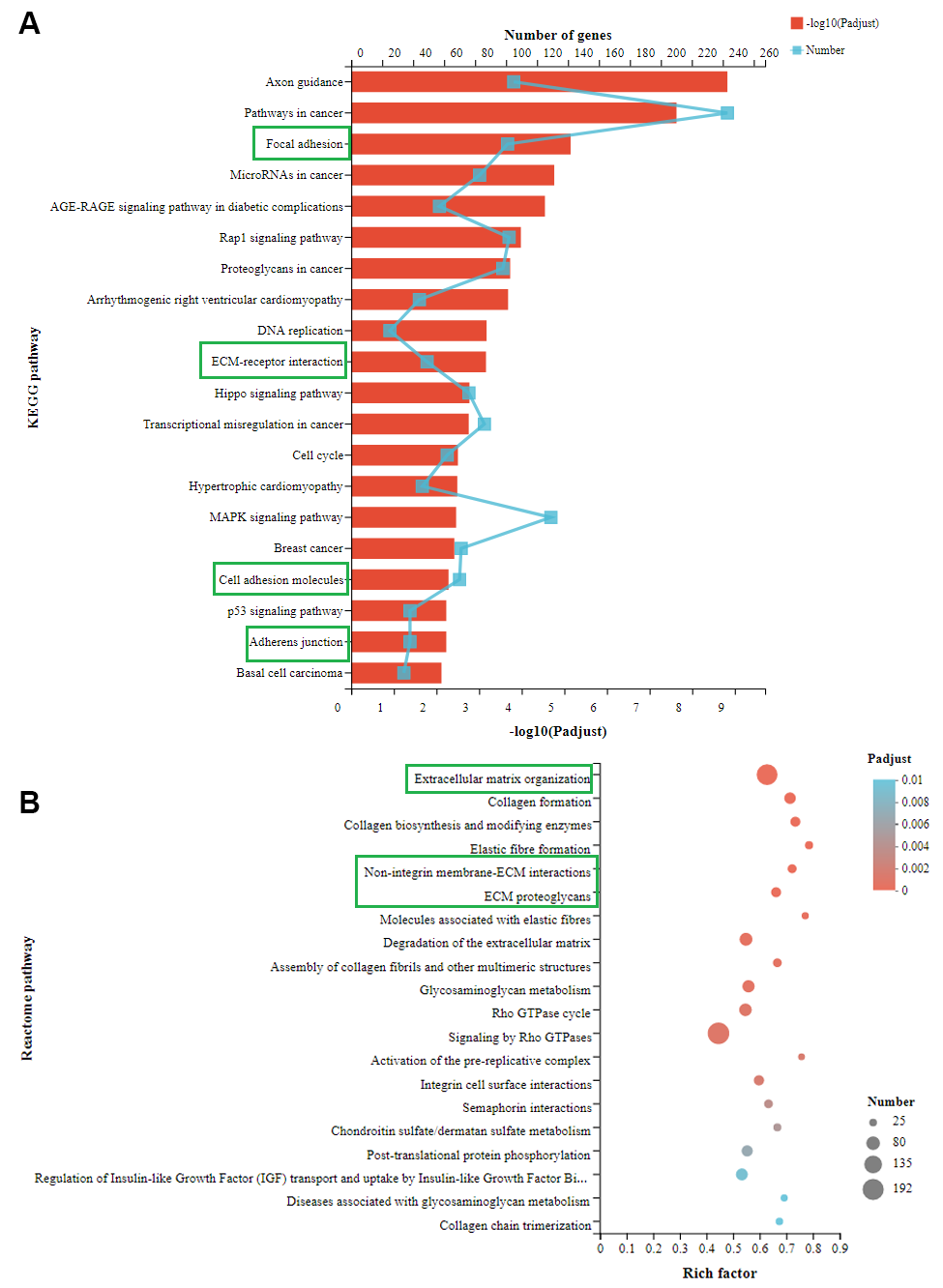
**Supplementary Figure S3. KEGG and Reactome pathways of IOSE80 and BMSCs** (A) KEGG enrichment analysis. The vertical coordinate represents the KEGG pathway, and the upper horizontal coordinate represents the number of genes/transcripts of the pathway, corresponding to different points on the polyline. The abscissa below represents the significance level of enrichment, corresponding to the length of the column. The smaller the *P*_adjust_ is, the larger the −log10 (*P*_adjust_) value is, and the KEGG pathway is enriched more significantly. The green box highlights pathways that are significantly enriched in cell adhesion, cell junction, and extracellular matrix. (B) Recatome enrichment analysis. The vertical axis represents the name of the Reactome path, and the horizontal axis represents the rich factor. The larger the rich factor is, the greater the degree of enrichment is. The size of the dot indicates the number of genes in this Reactome pathway, and the color of the dot corresponds to different *P*_adjust_ ranges. The green box highlights pathways that are significantly enriched in cell adhesion, cell junction, and extracellular matrix.


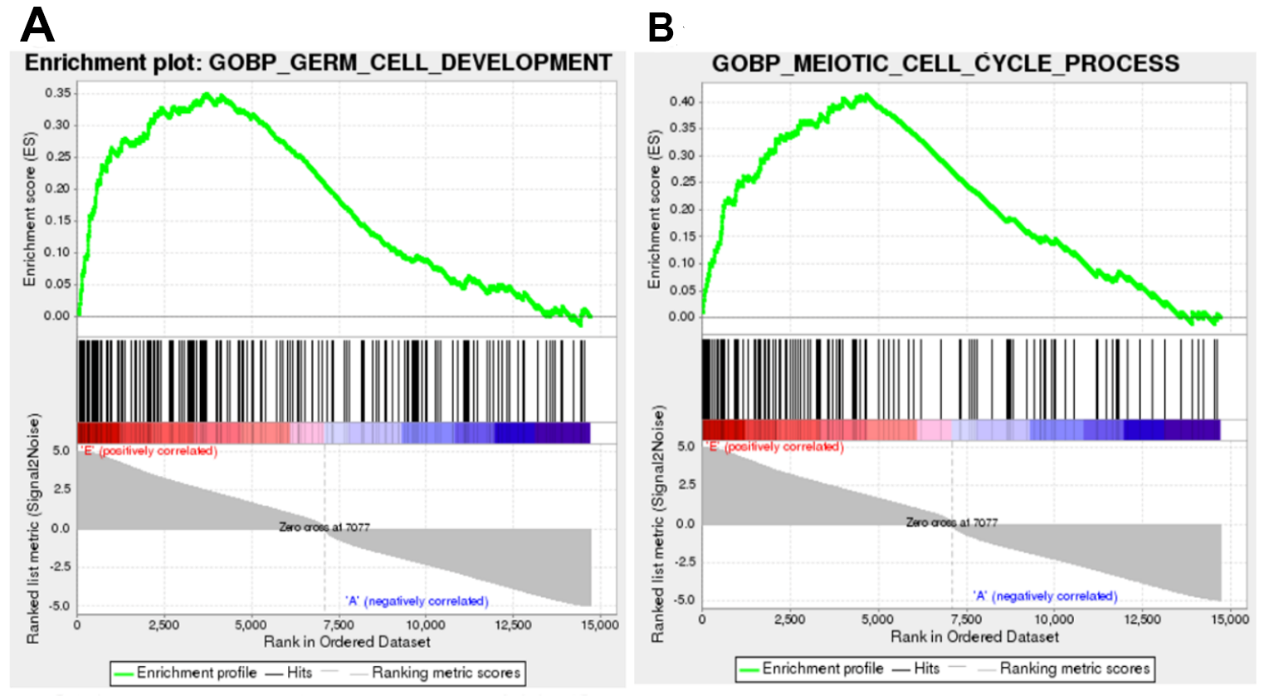


**Supplementary Figure S4.** **Gene Set Enrichment Analysis (GSEA)** (A,B) GSEA shows that functional genes from the “Germ cell development” (A) and “Meiotic cell cycle” (B) sets are enriched in IOSE80 cells compared to GV oocytes. The upper curve represents the change trend of the cumulative ES value, and the highest point is the ES value of the gene set. The middle vertical line indicates the position of the prior gene set in the sorted gene list, and the black line marks the position of each gene in the prior gene set in the sorted gene list. The lower heatmap and gray area map show the distribution of genes in the sequenced gene list. Heatmap: the genes corresponding to the red part are highly expressed in GV oocytes, and the genes corresponding to the blue part are highly expressed in IOSE80 cells; Area chart: the corresponding signal-to-noise ratio (Signal2noise, the calculation method of the previously selected sorting value) of each gene is displayed in a gray area chart.


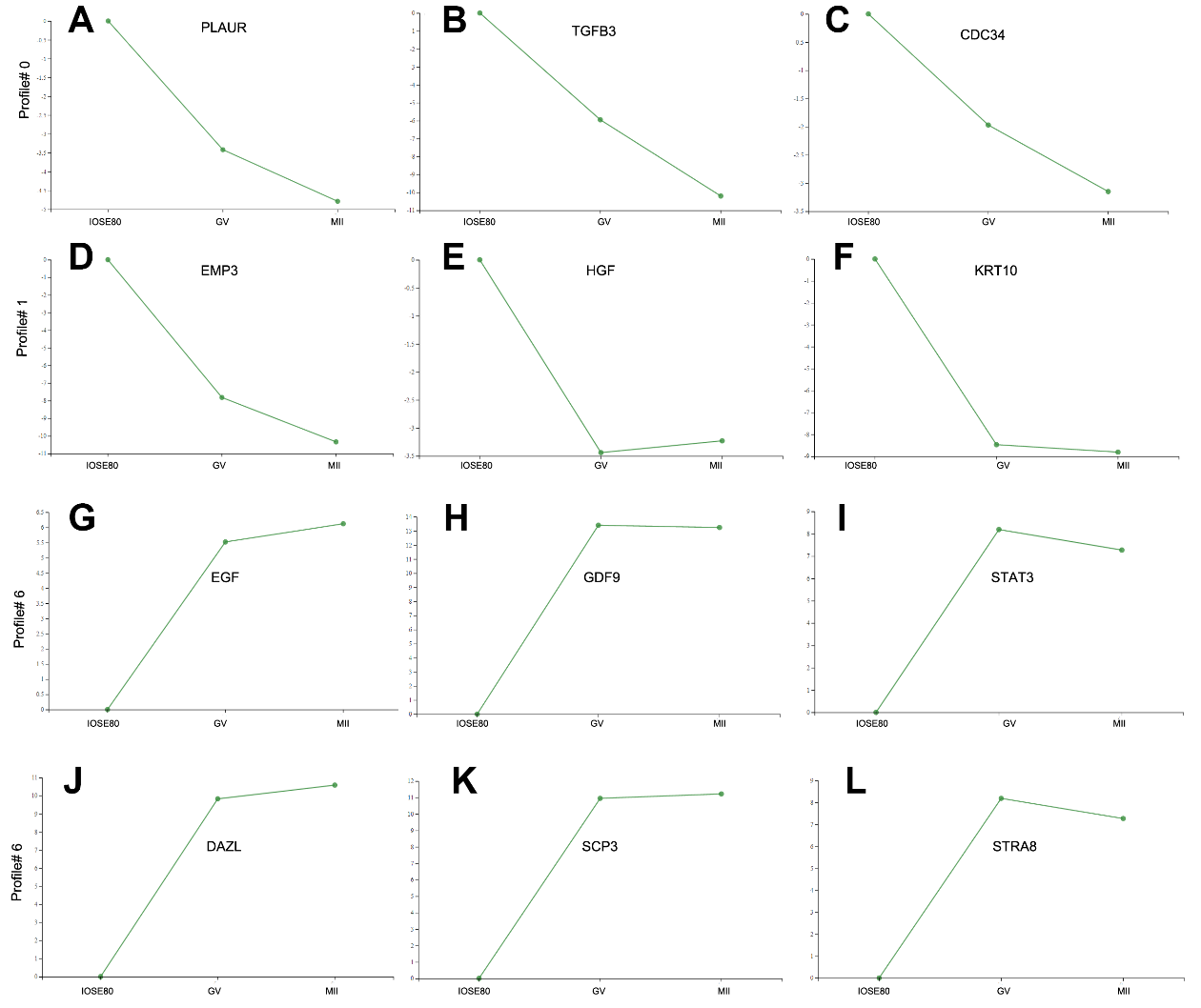
**Supplementary Figure S5. Time series expression trend analysis** (A−C) Three genes in the red module (Profile # 0) represent a decreasing expression trend. (D−F) Three genes in the red module (Profile # 1) represent a decreasing expression trend. (G−L) Six genes in the blue module (Profile # 6) represent the overall increased expression trend.


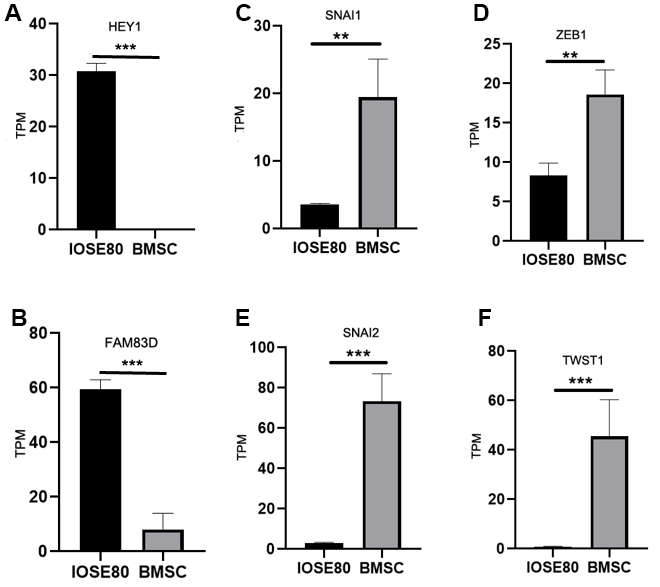
**Supplementary Figure S6. Differentially expressed genes in epithelial-mesenchymal transformation between IOSE80 cells and BMSCs**  HEY1 (A) and FAM83D (B) were highly expressed in IOSE80, while 18 genes (*SNAI1*, *ZEB1*, *SNAI2*, *TWST1*, *MSX1*, *etc*.) (C−F) were highly expressed in BMSCs. ***P*<0.01, ****P*<0.001.
